# Structure and genetic variability of golden mussel (*Limnoperna fortunei*) populations from Brazilian reservoirs

**DOI:** 10.1002/ece3.4941

**Published:** 2019-02-10

**Authors:** Pâmela Juliana Furlan‐Murari, Claudete de Fatima Ruas, Eduardo Augusto Ruas, Lucas Milanez Benício, Angela Maria Urrea‐Rojas, Angela Rocio Poveda‐Parra, Emerson Murari, Ed Christian Suzuki de Lima, Felipe Pinheiro de Souza, Nelson Mauricio Lopera‐Barrero

**Affiliations:** ^1^ Department of Animal Science Universidade Estadual de Londrina Londrina Brazil; ^2^ Department of Biology Universidade Estadual de Londrina Londrina Brazil; ^3^ Faculdade Arthur Thomas Londrina Brazil

**Keywords:** bioinvader, bivalve, environmental conservation, genetic analysis, microsatellite markers

## Abstract

The golden mussel, *Limnoperna fortunei* a highly invasive species in Brazil, has generated productive, economical, and biological impacts. To evaluate genetic structure and variability of *L. fortunei* populations present in fish farms in the reservoirs of Canoas I (CANFF), Rosana (ROSFF), and Capivara (CAPFF) (Paranapanema River, Paraná, Brazil), eight microsatellite loci were amplified. Five of those eight loci resulted in 38 alleles. The observed heterozygosity (Ho) was lower than the expected heterozygosity (He) in all populations, with a deviation from the Hardy–Weinberg equilibrium (HWE). The average value for the inbreeding coefficient (Fis) was positive and significative for all populations. There was higher genetic variability within populations than among them. The fixation index (Fst) showed a small genetic variability among these populations. The occurrence of gene flow was identified in all populations, along with the lack of a recent bottleneck effect. The clustering analysis yielded *K* = 2, with genetic similarity between the three populations. The results demonstrate low genetic structure and suggest a founding population with greater genetic variability (ROSFF). Our data point to the possible dispersal of *L. fortunei* aided by anthropic factors in the upstream direction. It was concluded that the three populations presented a unique genetic pool for Paranapanema River, with occurrence of gene flow.

## 1 INTRODUCTION

The golden mussel, *Limnoperna fortunei* (Dunker 1857), is a bivalve mollusk belonging to the family of sea mussels (Mytilidae, Mytiloida; Newell, 1969), which is able to inhabit both freshwater and brackish environments (Darrigran, 2002). Originally from Southeast Asia, the golden mussel exhibits a fast growth rate, short life cycle, a high osmoregulation capacity, and a planktonic larval stage (veliger) (Darrigran & Damborenea, 2009). The adult form can reach a size of 3–4 cm, with shells composed of two valves, and shows high filtration rates and facility to fecundate and form colonies, reaching a density of more than 150,000 individuals/m^2^ (Cataldo, Boltovskoy, Hermosa, & Canzi, 2005). Giordani (2013)also highlighted that this organism has a gland that secretes protein filaments, known as byssus, which allows its fixation on practically all types of natural or artificial substrates, being nowadays a matter of great concern for all sectors that develop activities associated with the use of water.

In Brazil, the occurrence of golden mussel was first reported at the end of 1998 and beginning of 1999, in the State of Rio Grande do Sul, in the Jacuí River delta, and in the Guaíba Lake basin, respectively (Mansur, Richinitti, & Santos, 1999; Mansur et al., 2003). Recent studies have demonstrated a wide territorial distribution of this species, which encompasses several South American river basins, such as the basins of rivers Paraguay, Paraná, Uruguay, La Plata (Pessotto & Nogueira, 2018), and even in water bodies in the Northeast region of Brazil, such as the São Francisco River Basin (Barbosa et al., 2016). In the Paranapanema River, a tributary of the Paraná River, the first occurrence of the species was recorded in 2006, in the Canoas I reservoir (Garcia, Orsi, Casimiro, & Kurcheski, 2009). Its dispersion occurs in several ways, involving different stages of its life cycle, both larvae and adult (MMA‐Ministério do Meio Ambiente, 2004).

The presence of the golden mussel in the Brazilian reservoirs has promoted significant environmental and economic impacts, which require frequent investment in maintenance and control. Damage to hydroelectric plants pipelines, pumps, turbines, boat hulls (Mansur et al., 2003), and net cages in fish farms (Oliveira, Ayroza, Castellani, Campos, & Mansur, 2014) are some of the main impacts caused by the spreading of this species. Considering all the detrimental effects caused by this species and its abundance in the invaded environments, it is crucial to find ways of controlling its populations that satisfactorily solve the incrustation problems without affecting the health of the local populations or causing environmental impacts. Genetic studies using molecular biology techniques might provide additional information about the golden mussel, as information about the dispersal pattern, population genetic structure, as well as the possible influence of environmental factors on these characteristics, enabling the development of new technologies that contribute to the control and understanding of the invasion mechanisms of the species (Ghabooli et al., 2013; Oliveira et al. 2014; Zhan et al., 2012). According to the MMA (2004), one of the greatest obstacles for the implementation of measures to control the dispersion of golden mussel is lack of genetic information.

Genetic structuring analyses performed in previous studies by Zhan et al. (2012) and Ghabooli et al. (2013) reinforce the idea of “jump” dispersal dynamics of *L. fortunei* in South America. According to the authors, human‐mediated transport of propagules (e.g., abandonment of lines and hooks, ballast water discharge, recreational activities) are important factors that contributed to the dispersal of the mussel along the La Plata and Parana River basins. Another invasive mollusk, Zebra mussel (*Dreissena polymorpha*), has shown low genetic differentiation among populations of the Great Lakes, North America (Astanei, Gosling, Wilson, & Powell, 2005). The authors also pointed out that ballast water discharge contributed to the invasion of this species. In this study, we test the hypothesis that the dispersal of *L. fortunei* in the Paranapanema River may occur mainly via anthropogenic factors, which would provide gene flow even among populations isolated by dams. Thus, we evaluate the genetic structure and variability of golden mussel (*L. fortunei*) populations in three reservoirs of the Paranapanema River, Paraná, collaborating to the understanding of invasion patterns in the assessed regions.

## 2 MATERIALS AND METHODS

### 2.1 Collected biological material

**Figure 1:**
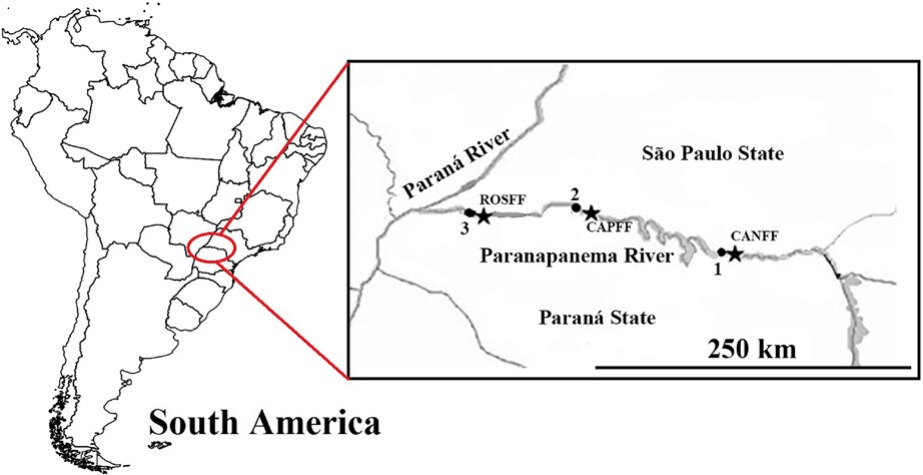
Location of the Canoas I (1), Capivara (2), and Rosana (3) reservoirs, indicated by the points, and the CANFF collection sites (22°56′25.63″S; 50°24′49.86″ W), CAPFF (22°41′17.16″S; 51°17′51.30″W), and ROSFF (22°39′25.20″S; 52°46′52.78″W), indicated by the stars, along the Paranapanema River

The samples were collected in December 2014 from fish farms located in three reservoirs of the Paranapanema River, State of Paraná (Figure 1):

Samples were collected from fish farms that use the open‐net tank production system to grow the Nile tilapia (*Oreochromis niloticus*). The mollusks were mechanically removed from the colonies, which remained fixated to the nets of the tanks. After removal, the mussels were placed in buckets and taken to nearby locations, where they were cleaned to remove any type of superficial dirt (slime, mud, etc.). The animals were subsequently kept for 2 hr in recipients containing ice (approximately 5°C) to induce numbness before they were killed. At this stage, they were cleaned again to remove dirt that remained between the mussels and were placed into labeled plastic bags. These bags were sealed and placed in thermos boxes filled with ice, before transporting them to the laboratory for analyses.

### 2.2 DNA extraction and quantification

DNA was extracted using a protocol based on that described by Lopera‐Barrero et al. (2008), with some modifications, in the Laboratory of Molecular Biology of the Nucleus of Study and Research in Aquaculture and Genetics, State University of Londrina (UEL). A total of 75 mussel samples (25 per fish farm) were analyzed. The mussel shell was opened, the animal was labeled, and the adductor muscle was removed with the help of tweezers by sectioning at the insertion region (basis). The sample was washed with absolute ethanol and then placed into a sterile microtube, where it was kept at room temperature for 10 min for the residual ethanol to evaporate. Next, the lysis solution (700 μl lysis buffer, 50 μl 20% SDS, and 15 μl 200 μg/ml proteinase K) was added to the samples, which were kept in a water bath at 50°C for 17 hr. The tubes were subsequently removed from the water bath and added with 700 μl of 5 M NaCl. The contents were then mixed by inversion before centrifugation at 11,270 *g* at 4°C for 10 min.

After centrifugation, 800 μl of the supernatant was removed from each sample and placed into a new sterile microtube, before the addition of 700 μl cold absolute ethanol for DNA precipitation. In order to increase the efficiency of the process, the microtubes were stored at −20°C for 2 hr. The samples were then centrifuged at 11,270 *g* at 4°C for 10 min. The ethanol (supernatant) was discarded, and the samples were dried at room temperature for 20 min. The samples were added with 35 μl TE (Tris/EDTA) and 5 μl RNAse (30 μg/ml) and kept in a water bath at 37°C for 40 min before storage at −20°C. DNA was quantified using a PICODROP^®^ spectrophotometer (Picodrop Limited, Hinxton, UK). The samples were diluted to a final concentration of 30 ng/μl. In order to assess DNA quality, an electrophoresis run in 1% agarose gel was conducted at 70 V for 1 hr.

### 2.3 DNA amplification and capillary electrophoresis

The amplification was performed at the Laboratory of Molecular Markers and Plant Cytogenetics at the Department of Biology, State University of Londrina (UEL). Eight primer pairs (Lf04, Lf06, Lf07, Lf19, Lf21, Lf22, Lf23, Lf38) previously developed for *L. fortune*i (Zhan et al., 2012) were tested in the analysis of the microsatellite regions (unique primers developed for this species until the present time). An indirect labeling with fluorophores was used for genotyping, using a system based on the addition of three primers to the PCR reaction, according to Schuelke (2000). In this method, a tail of the M13 sequence (TGTAAAACGACGGCCAGT) is added to the 5′ end of the primer. The amplification reactions were prepared with 4.5 μl GoTaq Green Master Mix (2X reaction buffer, pH 8.5, 1600 μM dNTP, and 3 mM MgCl_2_, Promega, Winchester‐USA), 0.08 μl 5 p.m. forward primer, 0.3 μl 5 p.m. reverse primer, 0.3 μl 5 p.m. M13 primer labeled with a fluorophore (Ned, Hex, 6‐Fam), 1 μl DNA (30 ng/μl), and 3.82 μl nuclease‐free water in a final volume of 10 μl.

The PCR reactions were performed in a PTC200 thermocycler (MJ Research, Massachusetts‐USA) using a gradient program. All eight primer pairs were tested, but only five of them were used (Lf06, Lf07, Lf21, Lf22, Lf23) as they presented the expected results: they were polymorphic and amplified the microsatellite alleles consistently and reproducibly. The respective PCR reactions using these five primer pairs were performed using the following program: 94°C for 5 min, followed by 30 cycles of 94°C for 30 s, respective AT (°C) for each primer for 30 s, and 72°C for 30 s, and a final extension at 72°C for 10 min. The final amplification product was subjected to an electrophoresis in the automatic sequencing machine 3500xL (Applied Biosystems, CA, USA) with the Formamide reactions Hi‐Di (8.8 μl), GeneScan LIZ600 Size Standard (0.2 μl), and DNA (PCR; 1 μl). The software Gene Marker v. 2.6 was used to calculate alleles size. It enables the determination of the allele size using the standard molecular weight marker, GeneScan™ 600‐LIZ^®^ Size Standard (Life Technologies, Califórnia, USA).

### 2.4 Statistical analyses

The results were displayed using data matrices and analyzed with the software FSTAT (Goudet 2005), to find the number of alleles per locus (Na), the allele richness (Ra), and the inbreeding coefficient (Fis). The number of effective alleles (Ae), observed (Ho) and expected heterozygosity (He), deviations from the Hardy–Weinberg equilibrium (HWE; *p* < 0.05; calculated by chi‐square test for Hardy–Weinberg equilibrium) for each locus, and genetic distance (Nei, 1978) were calculated using the software POPGENE (Yeh, Boyle, & Xiyan, 1999). Micro‐Checker 2.2.3 software (Van Oosterhout, Hutchinson, Wills, & Shipley, 2004) was used to detect possible null alleles such as Sutter bands that occur in a PCR reaction and hinder the reading of the Single Sequence Repeats (SSRs). BOTTLENECK software (Cornuet & Luikart, 1996) was used to verify whether the populations had undergone a recent bottleneck effect, taking into account the three evolutionary models for SSR loci (IAM—Infinite Allele Model, TPM—Two Phase Model, and SSM—Stepwise Mutation Model). ARLEQUIN software v. 3.11 (Excoffier, Laval, & Schneider, 2005) was used for the molecular variance analysis (AMOVA) and to determinate the allelic fixation index (Fst) and the linkage unbalance. The Wright definition (1978) was used to classify the Fst: values between 0.00 and 0.05, 0.051 and 0.15, 0.151 and 0.25, and >0.25 indicate small, moderate, high, and elevated genetic differentiation, respectively. BAYESASS (Wilson & Rannala, 2003) was employed to estimate gene flow using the Bayesian method. To identify the number (K) of genetically similar population clusters, we used the Structure v. 2.3.3 software (Hubisz, Falush, Stephens, & Pritchard, 2009) with a no‐admixture, length of burn‐in period of 50,000 and 500,000 repetitions of MCMC (Markov chain Monte Carlo), and 20 replicates per K, with K ranging from 1 to 6. The number of clusters was determined using the website Structure Harvester (Earl, 2012). Factorial correspondence analysis (FCA) based on allele frequencies was drawn by GENETIX software version 4.05 (Belkhir, Borsa, Chikhi, Raufaste, & Bonhomme, 2004).

## 3 RESULTS

Three of the eight loci tested (Lf04, Lf19, and Lf38) did not amplify or presented unspecific products, and were therefore removed from the analyses. The five loci used (Lf06, Lf07, Lf21, Lf22, and Lf23) were polymorphic and amplified consistent and reproducible microsatellite alleles, with the expected sizes between 57 bp (Lf23) and 291 bp (Lf21). In total, 38 alleles were detected for 75 individuals from the three natural populations of *L. fortunei*. The locus that presented the largest number of alleles (Na) was Lf06 (12 alleles), followed by Lf07 and Lf23 (7 alleles), and Lf21 and Lf22 (6 alleles). A low frequency of null alleles (5 alleles = 13.1%) was identified in the *L. fortunei* populations. Two loci showed null alleles in CANFF (Lf06 and Lf07), two in ROSFF (Lf06 and Lf22), and one (Lf06) in CAPFF.

Considering the results presented to verify the estimates of genetic diversity parameters of the five loci (Table 1), the average number of alleles (Na) per population ranged from 7.0 (ROSFF) to 4.6 (CANFF). The average values of allelic richness (Ra) differed among the populations, being the lowest for CANFF. The average number of effective alleles (Ae) varied between 2.032 (CANFF) and 3.282 (ROSFF), with an average value of 2.493 for the populations. The Ho values were lower than He in all populations, being significant (*p* < 0.05) for deviation from the HWE. The average Ho in each population ranged between 0.457 (ROSFF) and 0.275 (CANFF). The *p* values of Fis were compared to alpha of 0.05 after indicative adjusted nominal level (0.003), and the results presented a significant positive average value (*p* < 0.05) for all populations. Analysis using the Bottleneck program for the three mutation models indicated that in the populations, there has been no significant reduction in size in a short period of time. In other words, no recent bottleneck effect was observed. It was observed low genetic distance between the three populations (ROSFF × CANFF = 0.058; ROSFF × CAPFF = 0.049; and CANFF × CAPFF = 0.022).

**Table 1:** Estimation of genetic parameters of diversity at five microsatellite loci in the three *Limnoperna fortunei* populations (Pop)

*Primers*	Pop	Na	Ra	Ae	Ho	He	Fis
Lf06	CANFF	07	6.919	2.717	0.400	0.645*	0.385
	ROSFF	11	10.466	4.032	0.560	0.767*	0.274
	CAPFF	08	7.639	3.858	0.560	0.756*	0.263
Lf07	CANFF	05	5.000	3.085	0.368	0.694*	0.476
	ROSFF	06	6.000	4.060	0.600	0.773*	0.228
	CAPFF	05	4.950	2.469	0.500	0.610*	0.185
Lf21	CANFF	04	3.998	1.797	0.125	0.453*	0.728
	ROSFF	06	5.999	4.100	0.304	0.773*	0.612
	CAPFF	04	3.946	1.743	0.360	0.435*	0.176
Lf22	CANFF	03	2.946	1.331	0.280	0.254	−0.105
	ROSFF	06	5.464	1.846	0.280	0.468*	0.406
	CAPFF	04	3.913	1.357	0.208	0.269*	0.228
Lf23	CANFF	04	3.652	1.229	0.200	0.190	−0.053
	ROSFF	06	5.543	2.37	0.542	0.590	0.084
	CAPFF	04	3.890	1.398	0.320	0.291	−0.103
Average	CANFF	(23) 4.6	4.503	2.032	0.275	0.447*	0.391*
	CAPFF	(25) 5.0	4.867	2.165	0.390	0.472*	0.178*
	ROSFF	(35) 7.0	6.694	3.282	0.457	0.674*	0.327*

Notes

Ae: number of effective alleles; Fis: inbreeding coefficient (*p < 0.05); He: expected heterozygosity; Ho: observed heterozygosity; Na: number of alleles per population; Pop: Mussel populations; Ra: allele richness.

* Significance at 5% significance level for the Hardy–Weinberg exact test and for the inbreeding coefficient (Fis).

Using the analysis of molecular variance (AMOVA), a greater genetic variability was identified within than between the studied populations (Table 2). Likewise, the fixation index showed a small genetic differentiation (Fst) among them, according to the Wright definition (1978). Comparatively, CANFF × ROSFF presented the highest (0.050) and CANFF × CAPFF the smallest value (0.004). The Fst was significant (*p* < 0.05) in CANFF × ROSFF and CAPFF × ROSFF (0.046). Significant linkage unbalance associations (*p* = 0.05) were observed in the pairwise analyses performed for the evaluated loci in all populations. Four associations were observed in CANFF, five in ROSFF, and four in CAPFF.

**Table 2:** Analysis of molecular variance (AMOVA) and fixation indexes (Fst) for the three *Limnoperna fortunei* populations

Populations	Sources of variations	*df*	Sum of squares	Variance components	Percentage variation	Fst
CANFF × ROSFF	Between populations	1	0.980	0.003	5.02*	0.050
	Within populations	98	79.260	0.808	94.98	
	Total	99	80.240	0.812		
CANFF × CAPFF	Between populations	1	2.810	0.041	0.42^ns^	0.004
	Within populations	98	70.260	0.716	99.58	
	Total	99	73.070	0.758		
ROSFF × CAPFF	Between populations	1	3.550	0.050	4.60*	0.046
	Within populations	98	101.940	1.040	95.40	
	Total	99	105.490	1.090		

Notes

*df*: degrees of freedom; ns: not significant.

* Significance at 5% significance level (reported for 1,023 permutations).

In the Bayesian analyses for the number of migrants, the average percentage of nonmigrants was 0.788 for the CANFF population, 0.785 for ROSFF, and 0.766 for CAPFF. All the values are significant with confidence interval of 95%. Gene flow occurred in all populations, with an average rate of 0.110 in CANFF for migrants originating from ROSFF and 0.101 for those originating from CAPFF; 0.205 in ROSFF for migrants coming from CANFF and 0.010 for those coming from CAPFF, the latter being the lowest rate observed, and 0.020 in CAPFF for migrants originating from CANFF and 0.213 for those from ROSFF, the latter being the highest rate observed.

**Figure 2:**
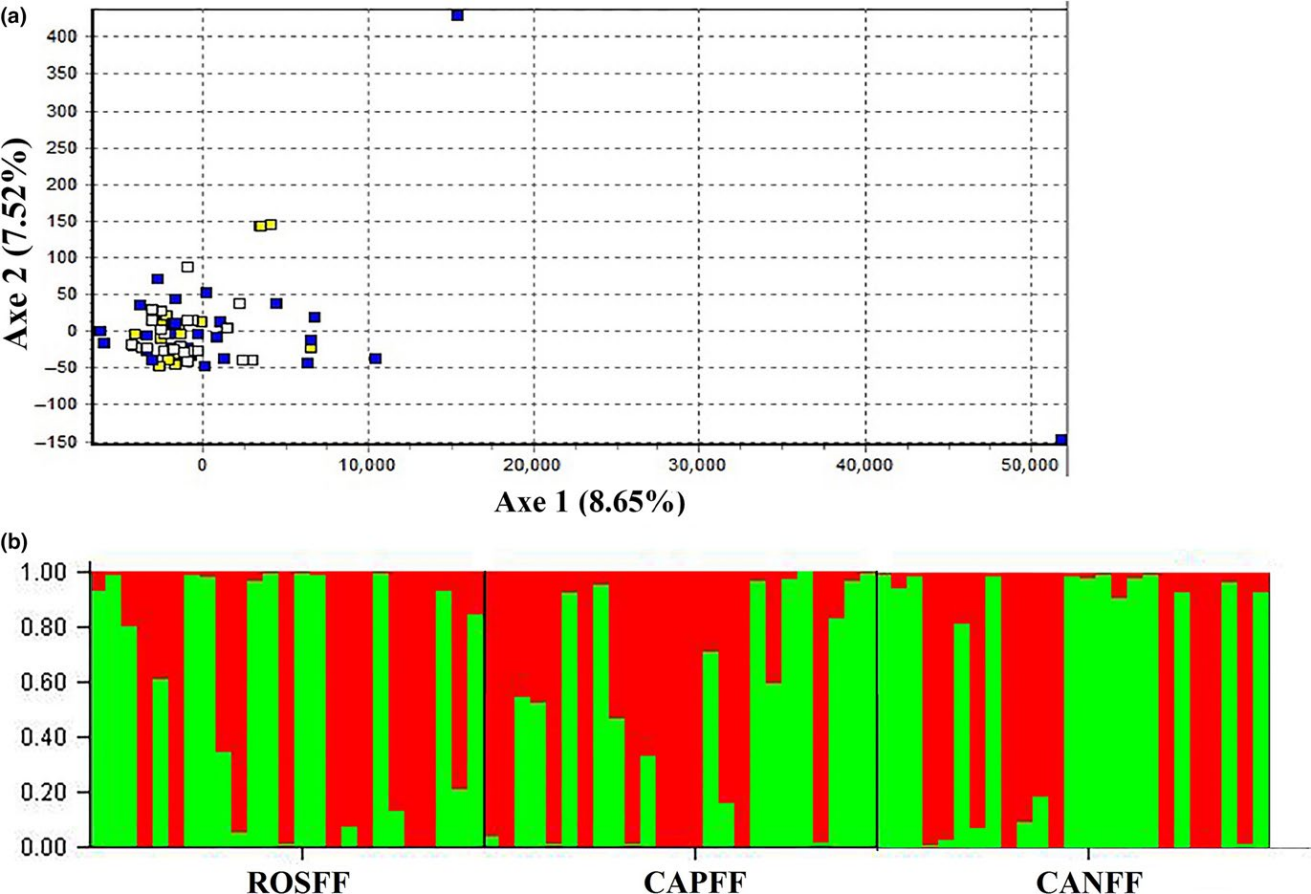
(a) Graphic representation of the first two factorial correspondence analysis (FCA), blue squares—ROSFF, white squares—CAPFF, and yellow squares—CANFF; (b) Clustering analysis of* (Limnoperna fortunei) *from Brazilian reservoirs

The factorial correspondence analysis (FCA) demonstrated overlapping individuals from the three populations (Figure 2a). This result corroborated by the clustering analysis (*K* = 2), with genetic similarity among all populations and greater proximity between CANFF and CAPFF (Figure 2b; Supporting Information Figure S1).

## 4 DISCUSSION

The five investigated loci were highly polymorphic and amplified consistent and reproducible microsatellites. No significant number of null alleles was found. This result might have been affected by the genetic proximity among populations, whose microsatellites flanking regions were conserved. On the other hand, the three loci that did not amplify or presented unspecific results demonstrated that the transferability of all primers developed by Zhan et al. (2012) may not be possible in different populations of *L. fortunei*. It is probable that the genetic differentiation of this species in new colonization sites has promoted changes in the flanking region of the primer, making it impossible the binding of these in the specific region. The low locus pairs in linkage disequilibrium suggest that they are not closely linked on the chromosomes (Kenchington, Patwary, Zouros, & Bird, 2006), and possibly did not affect the results.

The allele richness (Ra) and the effective alleles (Ae) were higher in ROSFF than in CANFF and CAPFF. The low Ae values compared to Ra indicate high frequency alleles. With the exception of ROSFF, the mean Ra values of CANFF and CAPFF were lower than those found by Zhan et al. (2012) and Ghabooli et al. (2013) in South American basins. The means of Ho and He indicated high genetic variability; however, there was a difference in heterozygosity (*p* < 0.05) across the three populations. This difference was characterized by a heterozygote deficit compared to HWE. Zhan et al. (2012) and Ghabooli et al. (2013) observed lower Ho values in the rivers Baía (0.2285), Corumbá (0.2214), and Itaipú (0.2802) in comparison with ROSFF and CAPFF. For CANFF, the average Ho (0.275) obtained here was similar than that found by these authors. Analyzing the bioinvasion of *Perna* in the Gulf of Mexico, Holland (2001) observed high genetic variability within populations and low interpopulation differentiation, a fact that was attributed to the conditions under which the invasion took place, such as multiple introduction events, introduction of a high number of larvae originating from several genetically distinct populations, or introduction of a high number or larvae from one single population with genetically diverse origins.

The heterozygote deficit can appear due to the presence of null alleles (Aung, Nguyen, Poompuang, & Kamonrat, 2010), the Wahlund effect, or a combination of both (Hatanaka, Henrique‐Silva, & Galetti, 2006), due to inbreeding (O'Connell & Wright, 1997), or as a consequence of the bottleneck effect (González‐Wangüemert et al., 2012). The analysis carried out using the Micro‐Checker software did not indicate the presence of a large number of null alleles, ruling out their interference. Likewise, the bottleneck effect, defined as a significant reduction in population size in a short period of time, did not affect the sampled populations according to the three evaluated mutation models. We believe that the Wahlund effect did not influence the results either, since the sampling was performed in geographically distant locations, which, in turn, reflected a habitat fragmentation caused by natural and artificial barriers. However, the low genetic structure verified by Structure and the low Fst values practically rule out the influence of the Wahlund effect on the heterozygote deficit. Therefore, the effect of an inbreeding process appears to be the most suitable explanation for the observed heterozygote deficit. Mating between related individuals has already been observed in bivalves (Li & Hedgecock, 1998), furthermore, two‐thirds of the *L. fortunei* population are composed by females, that is, the lower proportion of males increases the chance of recurrent inbreeding (Ricciardi, 1998; Zhan et al., 2012). In bivalves such as *Placopecten magellanicus*, Kenchington et al. (2006) also observed heterozygous deficits evidenced by Fis and related to inbreeding processes. According to the authors, the dispersion mechanism and spatial distribution of the young stages would favor groupings of related individuals, affecting the genetic structure and the Hardy–Weinberg equilibrium (HWE) in the populations.

The analyses of genetic structure showed consistent similarity among populations. The analysis of molecular variance (AMOVA) identified a higher genetic variability within than among the three populations, and the fixation index (Fst) indicated a low degree of differentiation among them. Likewise, the occurrence of gene flow was detected in all populations, along with a small genetic distance among them. Finally, corroborating our results, the FCA demonstrated an overlap in the distribution of genetic variability among populations, which was corroborated by clustering analysis (*K* = 2) that indicated two distinct genetic groups but with mix of individuals from all three populations (Figure 2). There are no previous studies of the genetic structure carried out in the sampled regions; however, existing genetic data in South American basins have revealed that even in geographically distant locations, such as Corumbá (Brazil), Quilmes, and San Fernando (Argentina), there may be no genetic structuring among mussel populations (Zhan et al., 2012).

In relation to the dispersion potential of *L. fortunei*, Pessotto & Nogueira (2018) found in the La Plata basin, more than 20 years after its introduction in this basin, high larval densities in all the main sub‐basins, with the species reaching lower latitudes in the upper Paraná sub‐basin, in a counter‐flux displacement greater than 2,000 km. Considering the history of the mussel invasion on the Paraná River, Avelar, Martim, and Vianna (2004) revealed the presence of the mussel in this river in 2002, downstream of the city of Rosana (22°32′56.9″S–53°2′48″W), characterizing the population as young and in full process of colonization. From this place to the meeting point of the Paraná River with the Paranapanema River, there is approximately 13 km. In this river, in a region close to the CANFF point, the mussel was first observed in 2006 (Garcia et al., 2009). Based on this information and taking into account that larvae and adults of *L. fortunei* present limited swimming abilities against currents, with its dispersion being carried out mainly by passive diffusion (Ricciardi, 1998), it can be concluded that the migration occurred upstream via anthropogenic factors. This hypothesis, which is supported by the dispersal dynamics by "jump", defended by Zhan et al. (2012) and Ghabooli et al. (2013) in the South American basins, is corroborated by structuring analyzes and gene flow. Based on the above, we conclude that the migration of *L. fortunei* through the Paranapanema River was mediated by one or more vectors, which allowed them to overcome the natural (current) and artificial (hydroelectric dam) barriers.

According to Holland (2001), when a population with a low genetic variability invades a heterogeneous and unknown habitat, the chances of fixation are small. On the other hand, for invading populations showing a high genetic variability, the fixation in a novel environment is easier. In the perspective of the present work, this leads us to consider a unique genetic pool for the whole extension of the sampled region (between ROSFF and CANFF), indicating that the colonization of the analyzed areas involved a large number of individuals. Therefore, it is presumed that the populations present in the reservoirs were formed from a founding population (ROSFF). Historically, the introduction and propagation of *L. fortunei* in South America started in Rio de la Plata (Argentina), following an upstream direction along the Uruguay, Paraguay, and Paraná basins (Ghabooli et al., 2013), where the Paranapanema River is located. In the present work, the hypothesis of ROSFF being the founding population is corroborated by the ascending migration of the mussel along the Paraná River, until reaching Rosana (Avelar et al., 2004), and later Canoas in 2006 (Garcia et al., 2009), already in the river Paranapanema. Reinforcing this hypothesis, greater genetic diversity was found in ROSFF, followed by CAPFF and CANFF, respectively, demonstrating a loss of diversity in the upstream direction. In addition, the significant genetic differentiation (Fst) of ROSFF in relation to CAPFF and CANFF can be explained by the hydrography of the region, since the Paraná River is widely used for waterway transportation, which facilitates the dispersion and entry of new individuals, such as observed in ROSFF, where two outliers individuals were found (Figure 2a). In the Paranapanema River, on the other hand, it only supports smaller vessels (fishing, recreation, and tourism, for example), which limits a wide dispersion of the mussel, explaining a smaller structuring between CAPFF and CANFF. Therefore, the existence of genetic flow in the downstream direction (CANFF to ROSFF) can be justified by the natural dispersion (via current), by migration of sessile larvae and/or by anthropogenic factors.

Darrigran and Damborenea (2009) considered the ballast water of vessels as one of the main vectors responsible for the introductions of molluscs in Latin America. Belz (2006) also highlighted several vectors, such as the abandonment of lines and hooks, the circulation of boats in rivers, or even the inland transport of small boats. It is likely that one or the combination of these factors may have favored the spread of *L. fortunei* along the Paranapanema River. Among the proposals that can support the control of the golden mussel, there is the importance of conducting new genetic research, collecting samples beyond the points already analyzed, still in the Paranapanema River, to clarify mechanisms of gene flow, genetic structure, and differentiation processes involved in the colonization of new habitats, besides the development of new specific molecular markers for *L. fortunei* from this region, considering the absence of amplification detected in some microsatellite primers. It is also considered important to carry out researches to identify the main vectors causing the dissemination of the mussel in the Paranapanema River, which will enable the implementation of inspection measures, readjustment, or even elimination of these vectors, as well as mapping of risk areas to avoid new colonization.

In conclusion, it was observed high variability with low genetic structure and occurrence of gene flow in both directions (upstream and downstream). The three populations presented a unique genetic pool for the entire stretch of the sampled river.

## CONFLICT OF INTEREST

The authors reported no potential conflict of interest.

## AUTHOR CONTRIBUTIONS

The study was coordinated by NMLB; samples collection was performed by PJFM, EM, and ARPP; FPS and PJFM extracted and quantified the DNA; LMB, CFR, and AMUR performed DNA amplification and capillary electrophoresis; statistical analyses were performed by EAR, CFR, and FPS; PJFM, NMLB, and ECSL performed the writing of the manuscript, and all authors contributed to editing the manuscript.

## Data Availability

Data about the 5 nuclear microsatellite markers in the 3 *Limnoperna fortunei* populations in Paranapanema River, locations of populations, and number of individuals is available in Dryad: https://doi.org/10.5061/dryad.b03475b.
