# Supplementary figures and images for: Structure and genetic variability of golden mussel (Limnoperna fortunei) populations from Brazilian reservoirs
Source: Ecol Evol. 2019 Feb 10;9(5):2706–14. doi: 10.1002/ece3.4941 (PMC6405496; doi:10.1002/ece3.4941)

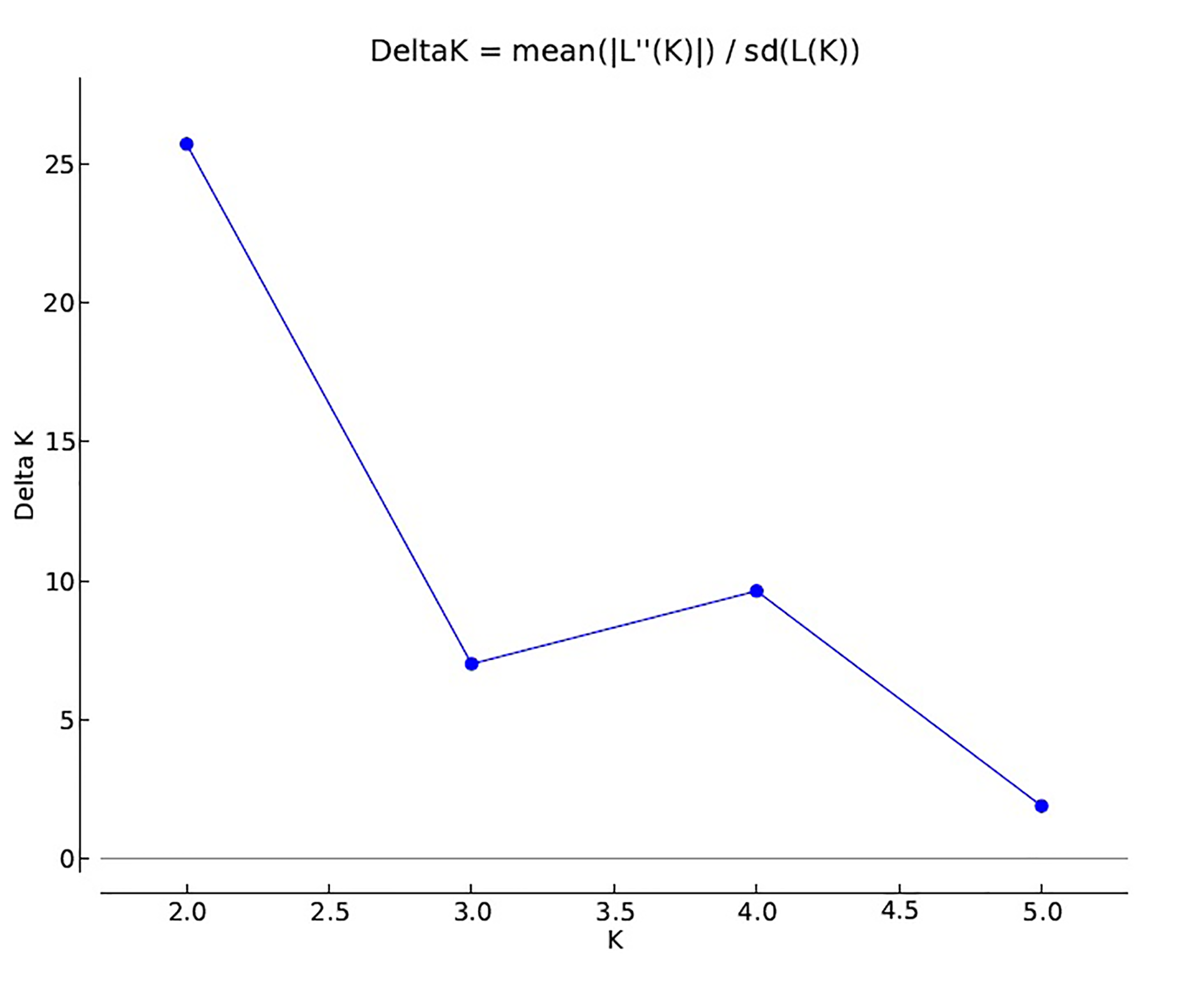

Supplement: Supplementary file 1 [file ECE3-9-2706-s001.tif]
